# How College Students Used Information From Institutions of Higher Education in the United States During COVID-19: Web-Based Cross-Sectional Survey Study

**DOI:** 10.2196/51292

**Published:** 2024-06-17

**Authors:** Emmanuel Peprah, Etornam Amesimeku, Brian Angulo, Himani Chhetri, Judy Fordjuoh, Christina Ruan, Cong Wang, John Patena, Dorice Vieira, Nessa Ryan, Chukwuemeka Iloegbu, Joyce Gyamfi, Jonathan Odumegwu

**Affiliations:** 1 Implementing Evidence-Based Interventions Through Engagement (ISEE) Lab Department of Global and Environmental Health New York University School of Global Public Health New York, NY United States; 2 Department of Biostatistics New York University School of Global Public Health New York, NY United States; 3 New York University Grossman School of Medicine New York, NY United States

**Keywords:** COVID-19, pandemic, public health, preventative, prevention, social distancing, masks, personal protective equipment, cross-sectional, surveys, higher learning, higher education, university students, information source, web-based information, health information, dissemination, awareness, information spread, young adults, social media, university, postsecondary, students, young adult, college, concern, worry, anxiety, perceptions

## Abstract

**Background:**

The start of the COVID-19 pandemic resulted in the implementation of nonpharmaceutical interventions by US institutions of higher education at an unprecedented level. During the backdrop of an emerging pandemic, younger adults (eg, college students) had an overall lower risk for severe outcomes for SARS-CoV-2, making this population a potential source of transmission for age groups with high susceptibility and negative health outcomes. We examine how college students’ level of concern for COVID-19 was influenced by different sources of information, their living status, income level, and other demographic identifiers and its association with prevention behavior change.

**Objective:**

We sought to examine the level of concern, defined as the extent to which the participant would take corrective action to mitigate contracting or spreading the virus (to family or friends) by using personal protective equipment such as a face mask, practicing social distancing, and following other public health recommendations, among college students during the COVID-19 pandemic.

**Methods:**

A cross-sectional, web-based survey was conducted in 2021 among 185 college students aged 18-41 years, with most living in New York City and the United States (n=134, 72.4%). Out of 185 college students, 94 provided their zip codes, with 51 of those college students indicating they lived in New York City areas. The participants completed the survey via a QR code. Study participants who did not complete the full survey or were not college students in any US college or university were excluded. Analyses were conducted using R (version 4.2.2; R Foundation for Statistical Computing).

**Results:**

Of 185 respondents participated in the study, 25 (13.5.%) used emails from their schools, 51 (27.6%) used mainstream media, and 109 (58.9%) used social media and other sources to obtain information about COVID-19. Of the 109 participants who learned about the pandemic from social media, 91 (83.5%) were concerned; however, only 63% (32/51) and 60% (15/25) of the participants who sourced information from mainstream media and their schools’ email, respectively, were concerned. Further, the participants who received information from social media and other sources were about 3 times more likely to be concerned about COVID-19 than participants who received information from the university via email (*P*=.036; OR=3.07, 95% CI: 1.06-8.83)..

**Conclusions:**

College students who received information from social media and other sources were more likely to be concerned about COVID-19 than students who received information from their school via emails.

## Introduction

With the emergence of the COVID-19 pandemic in March 2020 [1-3], many institutions of higher education (IHEs) in the United States proceeded to institute nonpharmaceutical interventions (NPIs) based on the Centers for Disease Control and Prevention recommendations to manage the spread of the SARS-CoV-2. NPIs included using face masks [4,5], remote learning, physical distancing, significantly limiting mass gatherings, travel restrictions, and implementing distance learning or remote classrooms [6]. During the process of developing new policies to mitigate viral spread on university and college campuses, institutional administrators at IHEs had to communicate new and evolving policies to students, faculty, and staff during an unprecedented pandemic. Furthermore, during this unparalleled time with few guidelines for operating large and small institutions, IHEs developed different communication strategies. For example, a cross-sectional study found that larger institutions (>10,000 students) were more likely than smaller institutions (≤5000 students) to provide information on how to make an appointment for COVID-19 testing [7]. Moreover, IHEs in the New York City metropolitan area did not provide information on COVID-19 testing in a uniform and comprehensive fashion, which could have contributed to public confusion on testing [7]. Within the context of the evolving pandemic, the communication of relevant information from IHEs was instrumental in adherence to NPI [8,9]. Older people relied on more sources of information about SARS-CoV-2 than younger adults, which resulted in the older population practicing more protective behaviors [10] due to their higher susceptibility to the virus [11-13]. Although younger individuals, especially college students, had an overall lower risk for severe health outcomes for SARS-CoV-2, this population could have been a significant source of transmission during the pandemic [6,14,15]. Few studies have explored how college students’ level of concern for COVID-19 is influenced by different sources of information, their living status, income level, and other demographic identifiers. We obtained data from college students (ie, undergraduates to doctorate students) via a web-based survey to investigate whether the level of concern for COVID-19 was influenced by the source of information, familial context, and other factors (eg, location, rural vs urban settings, and income level).

## Methods

### Sampling Procedure

This research represents a comprehensive cross-sectional investigation conducted in 2021, encompassing a diverse cohort of 185 college students aged 18-41 years. Using a meticulous approach, a nonprobabilistic sampling method was used to ensure comprehensive data collection from our target demographic of college students. Recognizing the practical constraints of time, resources, and accessibility, we strategically used a blend of convenience and snowball sampling methodologies to optimize our research outreach and efficiency.

To maximize our survey’s dissemination, we strategically placed flyers and posters in prominent campus locales such as student centers, libraries, and cafeterias, each featuring a convenient QR code for direct access to the survey. Additionally, to broaden our participant pool and reach segments of the college student community that might otherwise be underrepresented, a snowball sampling technique was implemented. This innovative approach encouraged participants to actively share the survey link with their peers, facilitating a more inclusive and diverse representation within our study cohort.

By integrating both convenience and snowball sampling strategies, we ensured a robust and inclusive data collection process, capturing a broad spectrum of perspectives and experiences within the college student population. This methodological approach not only bolstered the comprehensiveness of our findings but also facilitated a deeper understanding of the diverse dynamics at play within this demographic.

The study participants were students in the United States attending a college or university. The participants completed the survey via a QR code; individuals who did not complete the full survey or were not college students in any US IHE were excluded. Students who were not enrolled in an IHE at the time of the study were excluded. All respondents were informed about the purpose of the study, the voluntary nature of their participation, and the confidentiality of their responses. Informed consent was obtained electronically before the commencement of the survey.

A total of 390 college students responded to the survey, with 211 completed submissions, garnering a response rate of 54.1%. Following rigorous data cleaning procedures, we excluded 205 (52.7%) students due to ineligibility, not providing consent to participate, nonsensical answers, incomplete surveys, duplicates, being bots, contradictory responses, infeasible response values for age, or invalid IP address. This commendable response rate underscores the efficacy of our survey distribution methods and the relevance of our research to the participant population. It serves as a testament to the engagement and interest elicited by our study within the college student community, affirming the significance and impact of our findings.

### Outcome Variable

Participants were asked “How concerned are you about COVID-19 (also referred to as Coronavirus)?” Response options were on a 5-point Likert type, from “I’ve never heard of it” to “I’m freaked out” and “I’m wondering why the university is not doing enough.” For analysis, the responses were categorized into 2 groups: 4-5=concerned and 1-3=not concerned, which were defined as the extent to which the participant would take corrective action to mitigate contracting or spreading the virus (to family or friends) by using personal protective equipment such as a face mask, practicing social distancing, and following other public health recommendations. This study used an adaption of the survey that was used by Boden-Albala et al [16] at the Susan and Henry Samueli College of Health Sciences, University of California, Irvine.

### Predictor Variables

We collected other information including age, gender, ethnicity, location, family structure, occupation, income, location settings, and information sources from the participants. Information source was measured on 3 scales, email from the IHE, mainstream media, and social media or others (eg, Facebook, Instagram, and SMS text messages). Ethnicity was categorized into Hispanic and non-Hispanic. Family structure was measured as living alone, with family, and with others. Location and location settings were measured as within the United States (including zip code) or outside the United States and rural or urban, respectively. Sex was grouped into male, female, and others. Income level was measured, and for analysis, it was categorized into less than US $35,000 and US $35,000 and above.

### Data Analysis

Analyses were conducted using R (version 4.2.2; R Foundation for Statistical Computing). We conducted a descriptive analysis, using frequencies with percentages for categorical variables or means with SDs for continuous variables. We conducted a bivariate analysis between the level of concern and each of the predictor variables using the chi-square test and 2-tailed *t* test. We then iteratively used a multivariable logistic regression model to determine the predictors of the level of concern for COVID-19 from those variables in the bivariable analyses with *P*<.10 [17]. Statistical significance was assessed as *P*<.05.

### Sample Size Determination

We used the Fisher method to determine the sample size for the study, with a confidence level of 95%, an estimated 50% proportion of the students using social media as a source of information about COVID-19, and a margin of error of 5%. Thus, the minimum sample size for the study was 384.

### Ethical Considerations

This study received approval from the New York University’s Institutional Review Board (approval IRB-FY2020-4342), ensuring adherence to the highest ethical standards. All procedures were meticulously conducted in strict compliance with pertinent guidelines and regulations, with paramount importance placed on obtaining informed consent from all college-age participants (defined as aged 18 years and older) prior to their engagement in the web-based survey. Emphasizing privacy and confidentiality, the survey methodology facilitated the deidentification of data, and no personally identifiable information was gathered. Participants generously contributed their time and insights without any form of compensation for involvement in this research study.

## Results

### Participant Characteristics

As shown in Table 1, of the 185 participants, 127 (68.6%) of participants were non-Hispanic, 99 (53.5%) were female, and 162 (87.6%) were between 20 and 30 years of age. In addition, most participants, 134 (72.4%) lived in the United States, 149 (80.5%) lived with family or alone, and 133 (72.4%) were employed full-time or part-time. The majority of participants, 115 (62.2%), lived in urban areas, 107 (57.8%) were earning more than US $35,000, 109 (58.9%) sourced information about COVID-19 using social media, 51 (27.6%) sourced information through Mainstream media, and 25 (13.5%) sourced information through emails from their school.

**Table 1:** Characteristics of the participants, bivariable relationships between level of concern for COVID-19 and predictors, and covariates.

Variable		Total (n=185), n (%)	Concern, n (%)^a^			*P* value	
			Yes	No			
**Age range (years)**							.410
	20-30	162 (87.6)	120 (74.1)	42 (25.9)			
	31-40	18 (9.7)	13 (72.2)	5 (27.8)			
	41-51	5 (2.7)	5 (100)	0 (0)			
**Sex**							.051
	Male	73 (39.5)	48 (65.8)	25 (34.2)			
	Female	99 (53.5)	81 (81.8)	18 (18.2)			
	Others	13 (7)	9 (69.2)	4 (30.7)			
**Ethnicity**							.520
	Hispanic	58 (31.3)	41 (70.7)	17 (29.3)			
	Non-Hispanic	127 (68.6)	97 (76.4)	30 (23.6)			
**Geographical location**							.013
	Within the United States	134 (72.4)	107 (79.9)	27 (20.1)			
	Outside the United States	51 (27.6)	31 (60.1)	20 (39.2)			
**Family structure**							.261
	Living alone	72 (38.9)	49 (68.1)	23 (31.9)			
	Family	77 (41.6)	61 (79.2)	16 (20.8)			
	Others	36 (19.5)	28 (77.8)	8 (22.2)			
**Occupation**							.294
	Full-time employed	56 (30.3)	38 (67.9)	18 (32.1)			
	Part-time employed	77 (41.7)	58 (75.3)	19 (24.7)			
	Unemployed	52 (28.1)	42 (80.8)	10 (19.2)			
**Income (US $)**							.256
	<35,000	78 (42.2)	62 (79.5)	16 (20.5)			
	>35,000	107 (57.8)	76 (71)	31 (29)			
**Location settings**							.046
	Rural	70 (37.8)	46 (65.7)	24 (34.3)			
	Urban	115 (62.2)	92 (80)	23 (20)			
**Information source**							.003
	Email from the institute of higher education	25 (13.5)	15 (60)	10 (40)			
	Social media and others	109 (58.9)	91 (83.5)	18 (16.5)			
	Mainstream media	51 (27.6)	32 (62.7)	19 (37.3)			

^a^Percentages reported with the n value in the “Total” column as the denominator.

### Level of Concern

The percentage of participants concerned about the COVID-19 pandemic varied widely across sources of information. Of the 109 participants who learned about the pandemic from social media, 91 (83.5%) were concerned. In contrast, only 63% (32/51) and 60% (15/25) of the participants who sourced information about the pandemic through the mainstream media and their schools’ emails, respectively, were concerned (Table 1).

### Predictors

Multivariable analysis indicated that only the information source was a statistically significant predictor of the level of concern for the pandemic (Table 2). The participants who received information from social media and other sources were about 3 times more likely to be concerned about COVID-19 than participants who received information from their school via email (*P*=.036; odds ratio [OR] 3.07, 95% CI 1.06-8.83). In addition, the participants who sourced information through mainstream media were about 1.3 times more likely to be concerned about the pandemic than participants who received information from their school via emails (*P*=.665; OR 1.26, 95% CI 0.44-3.62); however, this relationship is not statistically significant.

**Table 2:** Multivariable logistics regression prediction for the level of concern for COVID-19.

Variable			OR^a^ (95% CI)		*P* value
**Sex (reference=male)**					
	Female	1.20 (0.47-3.03)		.693	
	Others	0.83 (0.22-3.56)		.788	
**Location (reference=within the United States)**					
	Outside the United States	0.50 (0.20-1.24)		.135	
**Occupation (reference=full-time employed)**					
	Part-time employed	1.37 (0.59-3.16)		.458	
	Unemployed	1.52 (0.59-4.06)		.392	
**Income (US $; reference=<35,000)**					
	>35,000	0.53 (0.24-1.12)		.101	
**Location settings (reference=rural)**					
	Urban	1.18 (0.52-2.60)		.682	
**Information source (reference=emails from the institute of higher education)**					
	Social media and others	3.07 (1.06-8.83)		.036^b^	
	Mainstream media	1.26 (0.44-3.62)		.665	

^a^OR: odds ratio.

^b^*P*<.05.

There was no significant difference in the level of concern for COVID-19 between female and male participants (*P*=.693; OR 1.20, 95% CI 0.47-3.03) and those living within and outside the United States (*P*=.135; OR 0.50, 95% CI 0.20-1.24). Furthermore, there was no significant difference between those who were part-time and full-time employed (*P*=.458; OR 1.37, 95% CI 0.59-3.16), those who were unemployed and full-time employed (*P*=.392; OR 1.52, 95% CI 0.59-4.06), those who had an income greater and less than US $35,000 (*P*=.101; OR 0.53, 95% CI 0.24-1.12), or those who live in urban and rural areas (*P*=.682; OR 1.18, 95% CI 0.52-2.60). The results indicate that information source was a significant predictor of concern for COVID-19, with participants receiving information from social media and other sources being more likely to be concerned about the pandemic. In addition, the Hosmer-Lemeshow goodness-of-fit test [18] indicates that the model fits the data well (*χ*^2^_8_=5.1; *P*=.77).

## Discussion

### Social Media Influence: Shaping COVID-19 Concern Among College Students

Overall, 185 students participated in this study. The majority of participants were non-Hispanic (n=127, 68.6%), female (n=99, 53.5%), living with family or alone (n=149, 80.5%), and located in urban areas (n=115, 62.2%; Table 1). Over half (n=109, 58.9%) of the participants reported that their primary information source was from social media, with the remaining using mainstream media (n=51, 27.6%) or their school’s emails (n=25, 13.5%). Most students reported being concerned about COVID-19. A participant’s location setting (*P*=.046), geographical location (*P*=.013), and COVID-19 information source (*P*=.003) were associated with a participant’s level of concern about COVID-19. Based on the multivariate regression model, the only statistically significant predictor of COVID-19 concern was the participants’ source of information.

Our findings revealed that students who relied on social media and other nontraditional sources for COVID-19 information exhibited significantly 3 times higher levels of concern compared to those who relied on information from their schools (*P*=.036; OR 3.07, 95% CI 1.06-8.83). The likelihood of concern for COVID-19 among students who relied on information from mainstream media was not significant different (*P*=.662; OR=1.26, 95% CI: 0.52-3.04) from that of those who relied on their schools. These results align with previous research indicating that social media can amplify the perception of risk during public health crises [19]. The heightened concern among students who use social media might be attributed to the nature of information dissemination on these platforms, where sensational and emotionally charged content tends to receive more engagement and visibility [20]. This can lead to an increased perception of risk, particularly in a rapidly evolving situation such as the COVID-19 pandemic. Additionally, the prevalence of misinformation on social media platforms could have contributed to higher levels of concern [19,21].

Remarkably, this study revealed no notable differences in the level of concern across various demographic factors such as sex, living arrangements, or employment status. This finding suggests that the informational sources individuals accessed played a pivotal role in shaping their perceptions of the COVID-19 pandemic. This highlights the importance of targeted and strategic public health communication efforts [22].

### Redefining Public Health Messaging: Leveraging Social Media for College Student Engagement

The prevalence of social media as a primary source of information among college students has significant implications for public health messaging. It suggests that IHEs may have overlooked valuable opportunities to engage with students effectively during the pandemic. Recognizing the increasing influence of social media, particularly among younger demographics, it becomes imperative for public health authorities and IHEs to harness these platforms for timely and accurate information dissemination [23].

Moreover, our findings emphasize the urgent need for collaborative efforts to combat misinformation on social media. This could involve partnerships between health authorities, educational institutions, and social media platforms to develop robust strategies aimed at enhancing digital literacy among college students. Empowering students with the skills to critically evaluate web-based information could prove instrumental in mitigating the spread of misinformation [24,25].

While communication channels used by IHEs remain vital, our findings suggest an imperative for these institutions to adapt and expand their approaches to effectively reach college students. The efficacy of social media in disseminating information and heightening awareness among college students is undeniable, presenting an opportunity for IHEs to enhance their engagement with students through these platforms.

Furthermore, this study suggests the pressing need for IHEs to address the challenge of misinformation on social media platforms. Given the heightened concern associated with social media use, ensuring the provision of accurate, timely, and reliable information is paramount. IHEs can play a pivotal role in this endeavor by potentially collaborating with social media platforms to verify and disseminate factual and beneficial information.

Our research contributes to the growing body of evidence highlighting the significance of information sources in shaping public health behaviors and concerns, particularly among young adults in academic settings. The findings advocate for a more integrated approach to communication strategies by IHEs, advocating for the leveraging of both traditional and social media channels to ensure effective information dissemination during health crises and beyond.

In contributing to the expanding literature on the influence of information sources on public health behaviors and perceptions during the COVID-19 pandemic, this study sheds light on the significant role of social media in shaping students’ concerns. This necessitates nuanced and multifaceted communication strategies that account for the diverse information-seeking behaviors of young adults. By addressing these complexities, public health interventions can better resonate with and effectively engage this demographic.

### Strengths and Limitations

Our data set and analysis had several limitations. First, our sample size is 185, which potentially limits the implications of our findings to the geographic area of New York City, where most of our respondents were located. However, several strengths include the collection of important participant demographic information (eg, ethnicity and sex) and other critical information (see the *Methods* section) that was essential to determine if any variables other than information source could also be a mitigating factor for the level of concern. We believe that our data established that the source of information was critical for the level of concern.

### Conclusions

The study findings highlight the profound impact of information sources on the level of concern among college students regarding COVID-19. Notably, our research revealed that students who relied on social media and other nontraditional sources for information were 3 times more likely to express heightened concern about COVID-19 compared to those who received information from their IHE via email. This stark disparity illuminates the influential role of social media in shaping perceptions and awareness during public health crises, emphasizing the need for IHEs to reassess and diversify their communication strategies, especially when engaging with the younger demographic.

## Abbreviations

IHE: institute of higher education
NPI: nonpharmaceutical intervention
OR: odds ratio

## Data Availability

The data sets generated and analyzed during this study are available from the corresponding author on reasonable request.
